# Special Issue “Advancement in Breast Diagnostic and Interventional Radiology”

**DOI:** 10.3390/diagnostics12010217

**Published:** 2022-01-17

**Authors:** Graziella Di Grezia

**Affiliations:** Scientific Responsible of Breast Screening Program, Radiology Division, “Criscuoli-Frieri” Hospital, 83054 Sant’ Angelo dei Lombardi, AV, Italy; graziella.digrezia@gmail.com

A multimodality approach in breast imaging is a unique solution to guarantee to the patient a complete diagnosis [1], the best therapeutical approach and an appropriate and personalized follow-up in relation to breast density [2].

In this Special Issue, we have presented different topics of the breast diagnosis, including new methods in experiments and information on less frequent cases (such as male breast carcinoma) [3,4].

The purpose of the issue is to analyze the state of the art of breast imaging for subspecialized radiologists, but also for non-dedicated medical staff, in order to strengthening the concept that breast imaging is a specific area that requires specific training [5].

Some authors published manuscript regarding digital breast tomosynthesis (DBT) in the analysis of nipple sparing. 

Even though magnetic resonance is the most used method in these cases, DBT-galactography increases the sensitivity and specificity of lesion detection by improving the image quality [6].

Another interesting article is about the radiomic analysis of features, which results from contrast-enhanced spectral mammography in the differential diagnosis between benign and malignant lesions [7,8].

In addition to radiomic analysis, some authors have defined a radiomics score based on multiregional diffusion-weighted imaging (DWI) and apparent diffusion coefficient (ADC) features combined with clinical factors for evaluating HER-2 2+ status of breast cancer [9].

DWI is used simultaneously with multi-slice (SMS) imaging (SMS rs-EPI) for the differentiation of breast malignant and benign lesions on a 3T MR scanner with a higher spatial resolution and slight reduction of scan time and with a better differentiation between malignant and benign lesions of the breast [10].

Experimental models of new methods such as MammoWave are one of the new efforts to improve breast imaging. It is very difficult to identify an option that can support methods with high diagnostic accuracy, However, experiments with results that are negative or not positive, as well as those that end in failure, are part of the development and allow research to continue [11].
